# Molecular Characteristics of *Enterococcus faecalis* and *Enterococcus faecium* from Bulk Tank Milk in Korea

**DOI:** 10.3390/ani11030661

**Published:** 2021-03-02

**Authors:** Sunghyun Yoon, Young Ju Lee

**Affiliations:** 1 Division of Microbiology, National Center for Toxicological Research, U.S. Food and Drug Administration, Jefferson, AR 72079, USA; sungyoon@knu.ac.kr; 2 College of Veterinary Medicine & Zoonoses Research Institute, Kyungpook National University, Daegu 41566, Korea

**Keywords:** bulk tank milk, *Enterococcus*, antimicrobial resistance, transposon, virulence

## Abstract

**Simple Summary:**

Enterococci can be an opportunistic pathogen in milk, which can easily disseminate antimicrobial resistance and virulence genes. The purpose of this study was to characterize and compare the enterococci isolates from samples of bulk tank milk obtained from four dairy companies in Korea to prevent the spread of pathogenic and antimicrobial-resistant enterococci in dairy companies. The results demonstrated various degrees of antimicrobial resistance and virulence-factor distribution in enterococci from bulk tank milk in Korea and support the assessment that pathogens from bulk tank milk can also become a reservoir for dissemination of antimicrobial resistance and virulence factors through cross-contamination processes.

**Abstract:**

Enterococci are considered to be environmental mastitis-causing pathogens that can easily spread antimicrobial resistance or virulence genes via horizontal transfer. In this study, the molecular characteristics of enterococci from bulk tank milk were investigated to assess the importance of dairy herd management. A total of 338 enterococci (305 *Enterococcus faecalis* and 33 *Enterococcus faecium*) were isolated from 1584 batches of bulk tank milk samples from 396 farms affiliated with four dairy companies in Korea, and significant differences (40.6–79.7%) (*p* < 0.05) in the prevalence of enterococci were observed in the samples from different companies. Enterococci showed the highest resistance to tetracycline (TET) (73.4%), followed by doxycycline (DOX) (49.7%) and erythromycin (ERY) (46.2%), while two enterococci isolates showed resistance to vancomycin (VAN). Among 146 tetracycline (TET) and ERY-resistant enterococci, each 50 (19.4%) enterococci carried combination-resistance and transposon gene types *erm*(B) + *tet*(M) + *IntTn* and *erm*(B) + *tet*(L) + *tet*(M) + *IntTn*, respectively. The virulence genes such as *ace* (99.0%), *efaA* (97.7%), *cad1* (95.7%), and *gelE* (85.9%) were highly conserved in *E. faecalis* and significantly predominated over *E. faecium* (*p* < 0.001). Our results indicate that pathogens from bulk tank milk can also become a reservoir for the dissemination of antimicrobial resistance and virulence factors through cross-contamination processes.

## 1. Introduction

Enterococci are normal flora commonly found in the gastrointestinal tracts of humans and animals but are environmental mastitis-causing pathogens. In particular, *Enterococcus faecalis* (*E. faecalis*) and *Enterococcus faecium* (*E. faecium*) are the major species that account for approximately 90% of enterococci causing inframammary infection and are generally isolated from infected udders and dairy environments [1]. The udders of cattle are the primary reservoir of enterococci, and their pathogens can cause infections among quarters and cows during the milking process. The best way to treat mastitis is through the affected udder compartment, but the efficacy of antimicrobials can be limited due to the emergence of resistance against some antimicrobials [1].

Enterococci often acquire resistance by acquiring new genes with antimicrobial resistance on plasmids or transposons that can cross species and genera. Recently, resistance against tetracycline (TET) and erythromycin (ERY) has been reported in enterococci from many food-producing animals [2,3], and the presence of resistance genes against TET and ERY has been reported to be associated with resistance in general [3,4]. In the last few decades, clinical and subclinical mastitis have been successfully treated with TET and ERY, which are the most widely marketed antimicrobials in Korea [5], but increasing resistance to these antimicrobials from animal isolates continues to be reported [4,5,6,7].

The virulence of enterococci can enhance enterococcal infections and contribute to their potential pathogenesis [8]. Many researchers have reported that several virulence factors in enterococci may be involved in disease severity or exacerbation in humans and animals [8,9,10]. However, the direct relationship between virulence factors and the onset of diseases in cattle, such as clinical mastitis, have not been elucidated. Moreover, relatively little is known about the prevalence of virulence factors in enterococci from dairy products in Korea.

Antimicrobial resistance and virulence genes can be carried on mobile genetic elements [11]. Therefore, enterococci in milk can contribute to the spread of potentially pathogenic, antimicrobial-resistant strains to humans through food. The purpose of the present study was to compare the prevalence and distribution of antimicrobial resistance and virulence factors in enterococci isolates recovered from milk samples obtained from four dairy companies and to prevent the spread of pathogenic, antimicrobial-resistant enterococci in dairy companies.

## 2. Materials and Methods

### 2.1. Sample Collection and Bacterial Isolation

A total of 1584 batches of bulk tank milk samples were collected from 396 farms managed by four dairy companies in Korea. Milk samples were aseptically collected twice each in the summer and winter seasons. The isolation and identification of *Enterococcus* spp. were performed following the standard microbiological protocols published by the Ministry of Food and Drug Safety (Korea) (2018) [12]. Briefly, 1 mL of milk sample was cultured in 9 mL of buffered peptone water (BPW; BD Biosciences, San Jose, CA, USA). Then, pre-enriched BPW was mixed with enterococcosel broth (BD Biosciences) at a 1:10 ratio and incubated at 37 °C for 18–24 h. Each medium was streaked onto enterococcosel agar (BD Biosciences), and the formation of *E. faecalis* and *E. faecium* was confirmed using polymerase chain reaction (PCR) with specific primers such as *ddl_E. faecalis_* and *ddl_E. faecium_* as previously described [13].

### 2.2. Antimicrobial Susceptibility Testing

Based on the Clinical and Laboratory Standards Institute (Korea) guidelines, isolates were investigated for antimicrobial resistance using the disc diffusion test with the following discs (BD Biosciences): penicillin (PEN, 10 units), ampicillin (AMP, 10 μg), vancomycin (VAN, 30 μg), chloramphenicol (CHL, 30 μg), ciprofloxacin (CIP, 5 μg), ERY (15 μg), rifampin (RIF, 5 μg), TET (30 μg), and doxycycline (DOX, 30 μg) [14]. *E. faecalis* ATCC 29212 was used as the quality control. Multidrug resistance (MDR) was defined as acquired non-susceptibility to at least one agent in three or more antimicrobial classes [15].

### 2.3. Detection of Antimicrobial Resistance, Transposons, and Virulence Genes

The isolates were tested for the presence of the TET-resistance genes (*tet*(L), *tet*(M), and *tet*(O)) [2], E-resistance genes (*erm*(A), *erm*(B), and *mef*) [16], and Tn*916/1545*-like and Tn*5397*-like transposon genes (*Int-Tn* and *tndX*, respectively) [2,17] by PCR using primers previously described. Virulence genes such as *ace* (collagen-binding protein)*, asa1* (aggregation substance), *cad1* (sex pheromone), *cylA* (cytolysin activator), *efaA* (cell wall-associated protein involved in immune evasion), *esp* (enterococcal surface protein), *gelE* (gelatinase), and *hyl* (glycoside hydrolase) were also determined by PCR as previously described [4,18].

### 2.4. Statistical Analysis

Statistical analyses were performed using SPSS version 25 (IBM Corp., Armonk, NY, USA). Analyses of the differences in enterococci prevalence, antimicrobial resistance, antimicrobial-resistant genes, and virulence genes in milk samples from different companies, were conducted using Chi-square tests. Differences were considered significant at *p* < 0.05.

## 3. Results

### 3.1. Prevalence of Enterococci

**Figure 1:**
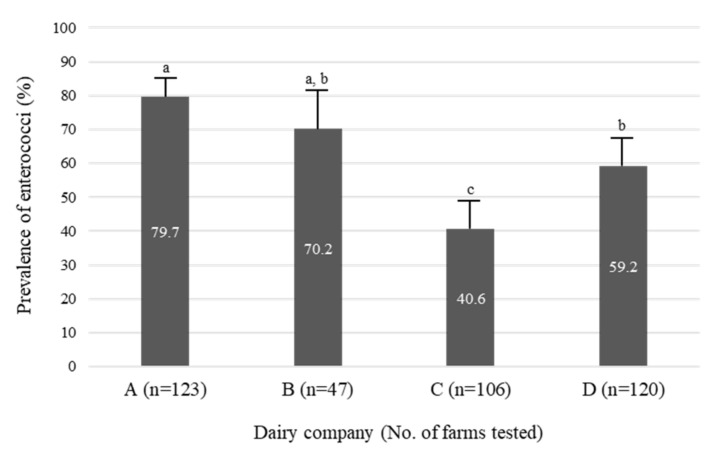
Prevalence of *Enterococcus faecalis* and *Enterococcus faecium* in bulk tank milk samples from four dairy companies. Values not sharing a common subscript letter (a, b, c) are statiscally different (*p* < 0.05).

The prevalence of enterococci in bulk tank milk samples from four dairy companies is shown in Figure 1. The prevalence of *E. faecalis* and *E. faecium* in samples from the four companies varied from 40.6 to 79.7%. Samples from company A had a significantly higher prevalence of *E. faecalis* and *E. faecium* than samples from the other companies (*p* < 0.05).

### 3.2. Distribution of Antimicrobial Resistance

The antimicrobial resistance of 338 isolates of enterococci (305 *E. faecalis* and 33 *E. faecium*) taken from samples of bulk tank milk from four dairy companies is shown in Table 1. Enterococci showed high rates of resistance to TET (73.4%), followed by DOX (49.7%) and ERY (46.2%). Moreover, resistance to CHL, CIP, ERY, TET, PEN, and VAN showed significant differences depending on the source company (*p* < 0.05). In particular, enterococci isolates from samples of bulk tank milk from company C showed significantly higher rates of resistance to C (33 isolates, 56.9%), ERY (41 isolates, 70.7%), and TET (51 isolates, 87.9%) than isolates from the samples from other companies (*p* < 0.05); two enterococci isolates from samples from company B showed resistance to VAN.

### 3.3. Distribution of MDR Isolates

**Figure 2:**
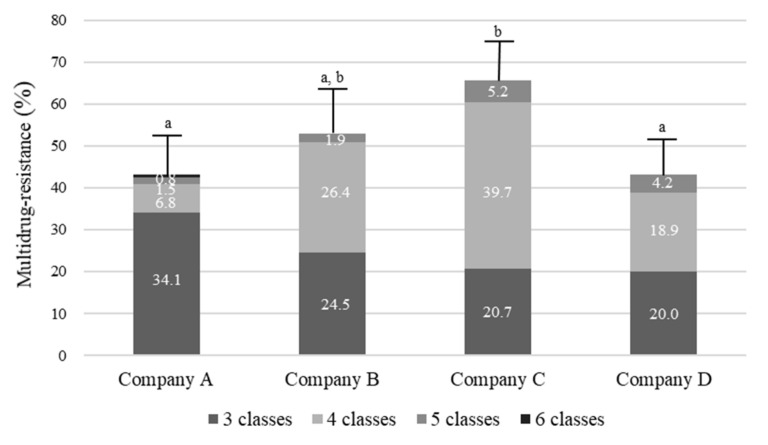
Distribution of multidrug resistant *Enterococcus faecalis* and *Enterococcus faecium* in bulk tank milk from four dairy companies. Values not sharing a common subscript letter (a,b) are statistically different (*p* < 0.05).

The distribution of 164 MDR enterococci in samples of bulk tank milk from four dairy companies is shown in Figure 2. The prevalence of MDR was also significantly different depending on the source company (*p* < 0.05). In particular, MDR (70.4%) in enterococci isolated from samples obtained from company C showed a significantly higher rate than isolates from the samples obtained from other companies (*p* < 0.05). Although one isolate of enterococci (0.8%) from company A showed MDR against six antimicrobial classes, 45 isolates (34.1%) and 19 isolates (20.0%) from companies A and D, respectively, showed the highest MDR against three classes. Moreover, 14 isolates (26.4%) and 23 isolates (39.7%) from companies B and C, respectively, showed the highest MDR against four classes.

### 3.4. Distribution of Antimicrobial Resistance and Transposon Genes in TET- and ERY-Resistant Enterococci

The distribution of resistance and transposon genes in 146 TET- and ERY-resistant enterococci isolated from samples of bulk tank milk from four dairy companies is shown in Table 2. Although one isolate of TET- and ERY-resistant enterococci showed none of the genes, 145 (99.3%) enterococci isolates carried various types of resistance genes. In particular, the most prevalent types, *erm*(B) *+ tet*(M) *+ IntTn* (50 isolates, 19.4%), showed significant predominance in samples from companies A and C, while *erm*(B) *+ tet*(L) *+ tet*(M) *+ IntTn* (50 isolates, 19.4%) showed significant prevalence in samples from company D (*p* < 0.05).

### 3.5. Distribution of Virulence Genes

The distribution of virulence genes in 305 *E. faecalis* and 33 *E. faecium* isolates is shown in Table 3. The most prevalent virulence gene in the *E. faecalis* isolates was *ace* (302 isolates, 99.0%), followed by *efaA* (298 isolates, 97.7%), *cad1* (292 isolates, 95.7%), *gelE* (262 isolates, 85.9%), *asa1* (162 isolates, 53.1%), *esp* (46 isolates, 15.1%), and *cylA* (20 isolates, 6.6%). In particular, the prevalence of *asa1, cylA,* and *gelE* genes was significantly different depending on the source company (*p* < 0.05). *E. faecium* isolates showed the highest prevalence of the *cad1* gene (81.8%). Although *ace* (4 isolates, 12.1%), *efaA* (4 isolates, 12.1%), *asa1* (3 isolates, 9.1%), *gelE* (2 isolates, 6.1%), and *esp* (1 isolate, 3.0%) were conserved in a small number of *E. faecium* isolates, all tested virulence genes showed no significant differences between source companies (*p* < 0.05). Moreover, the prevalence of virulence genes such as *ace, asa1*, *cad1*, *efa1*, and *gelE* was significantly higher in *E. faecalis* isolates than in *E. faecium* isolates (*p* < 0.001). None of the *E. faecalis* and *E. faecium* isolates carried the *hyl* gene.

## 4. Discussion

Enterococci can be an opportunistic pathogen in milk that leads to clinical or subclinical mastitis [19]. Since enterococci can easily spread antimicrobial-resistance or virulence genes via horizontal transfer, the presence of enterococci in milk can enhance the emergence of MDR strains, which can eventually affect the choice of drug [19]. In this study, the prevalence of *E. faecalis* and *E. faecium* differed significantly in bulk tank milk samples from four dairy companies. The presence of environmental pathogens such as enterococci in bulk tank milk is associated with poor hygiene. Good hygienic practices in dairy livestock management are important in reducing udder contamination from microbial sources such as the environment, feces, and slurry [20].

In this study, enterococci showed a high rate of resistance to the tetracyclines TET and DOX and the macrolide ERY. Although TET has been banned as a feed additive to reduce antimicrobial resistance in Korea since 2009, tetracyclines such as chlortetracycline calcium, chlortetracycline HCL, oxytetracycline dihydrate, and oxytetracycline HCL continue to be used for the treatment of bovine mastitis and are reported to be the most heavily used antimicrobial agents in dairy fields in Korea [5]. Although ERY is rarely marketed to the Korean dairy industry, the emergence of resistance to ERY in Korea has been previously reported [5,21] and is likely linked to other macrolides such as tylosin. Tylosin is widely used for the treatment of diseases such as streptococcal mastitis in Korea [19]. Moreover, enterococci in samples from company C showed significantly higher MDR, including resistance to TET and ERY, than enterococci isolates in samples from other companies, although their prevalence was the lowest among the samples from all four dairy companies. The results may reflect the use of antimicrobial treatments by dairy companies. Given that misused and overused antimicrobials can contribute to the emergence of antimicrobial-resistant strains and MDR development in the milk production system, further investigation to clarify the association will be needed.

In this study, one *E. faecalis* and one *E. faecium* isolate showed resistance to VAN. Although the appearance of vancomycin-resistant enterococci (VRE) in Korea was first reported from hospital infections in 1992 [22], VRE continues to be reported in environmental samples such as sewage, animal waste, and meat and milk products worldwide [23,24,25,26]. VAN is considered to be one of the last resorts for treatment of gram-positive bacterial infections in humans [27] but can be easily transferred through plasmids or transposons, acquiring resistance genes that enable bacteria to block cell-wall formation [28]. The persistence of VRE in milk products is considered to play an important role in human colonization and infection and should be continuously monitored in the dairy production system.

CHL-resistant enterococci usually presented with co-resistance to other antimicrobials such as linezolid or ERY because of resistance genes located adjacent to other antimicrobial-resistant genes in the same plasmid [29,30]. In this study, the prevalence of CHL-resistant enterococci was 25.4%; the high rate of ERY resistance (46.2%) may be associated with the genetic environment sharing similar antimicrobial-resistant genes.

In particular, RIF-resistant enterococci were detected in samples from all four companies (71 isolates, 21.0%). RIF is commonly used in combination with other antimicrobials for synergetic efficacy in reducing pathogens that remain during antimicrobial therapy [31]. Thus, RIF should be used with caution as it can limit treatment options for MDR pathogens [31].

Many researchers have also reported that genes associated with ERY and TET resistance are easily transferred via conjugative transposons [2,4,17]. In this study, the presence of TET-resistance genes (*tet*(L), *tet*(M), and *tet*(O))*,* macrolide-resistance genes (*erm*(A), *erm*(B), and *mef*), and transposon genes (*Int-Tn* and *tndX*) were simultaneously compared. The *tet*(L) and *tet*(M) genes were more prevalent than the *tet*(O) gene, while the *erm*(B) gene was more prevalent than *erm(A).* The *tet*(L)*, tet*(M), and *erm*(B) genes have been reported to be the genes most frequently found in enterococci isolates from humans and animals [3,18]. However, the distribution of combination gene types was significantly different depending on the company from which the samples were sourced. The combination of *erm*(B)*, tet(M), and IntTn* genes, which is one of the two most prevalent types, showed significant predominance in samples from companies A and C, while the combination of *erm*(B)*, tet*(L)*, tet*(M)*, and IntTn* genes, which is other of the two most prevalent types, showed significant prevalence in samples from company D. The simultaneous presence of combined *erm*(B)*, tet*(M), and *IntTn* genes and combined *tet*(L), *tet*(M)*,* and *IntTn* genes has already been reported in *E. faecalis* in chickens in Korea [2,4]. Isolates with transposon family Tn*916* (*IntTn*) have been previously reported; they harbor antimicrobial-resistance genes such as *tet* and *erm* on active mobile genetic elements facilitating gene transfer by conjugation, which can lead to multi-antimicrobial resistance [32]. In this study, isolates from samples obtained from three of the four dairy companies showed a significantly high prevalence of the transposon gene, *IntTn*, and other antimicrobial-resistance genes, which is problematic. Thus, the distribution of antimicrobial-resistance and transposon genes should be simultaneously monitored, and continuous surveillance will be important for the prevention of the emergence of MDR strains in the dairy industry.

The virulence factors of enterococci can contribute to the severity of pathogenesis. In this study, the virulence genes such as *ace* (99.0%), *efaA* (97.7%), *cad1* (95.7%), *gelE* (85.9%), and *asa1* (53.1%) were highly conserved in *E. faecalis*. Yang et al. (2019) also reported similar results with *E. faecalis* isolates from mastitic milk samples in China [3]. The collagen-binding protein, *ace*, promotes collagen binding in the extracellular matrix. The endocarditis-specific antigen, *efaA*, is associated with biofilm production. These genes are the virulence factors that promote colonization of enterococci in host tissues. Colonization does not necessarily induce pathogenicity but can be harmful when combined with other virulence factors or antimicrobial-resistance genes [33]. The sex pheromone gene, *cad1*, is reported to be the gene that facilitates conjugation. The presence of this gene can accelerate gene transfer, which is considered to be a public health risk [9]. The gelatinase gene, *gelE*, is referred to as one of the determinant virulence factors in enterococci and is capable of hydrolyzing bioactive peptides, collagens, elastin, and gelatin [34]. However, the prevalence of *asa1, cylA,* and *gelE* genes varied significantly in samples from the companies in this study. This may result from dissemination of the virulence genes within the same environment through horizontal transfer. *E. faecium* isolates showed the *cad1* (81.8%) to be the most prevalent virulence gene, but none of the genes showed significant differences in samples from the companies studied. In general, *E. faecalis* is reported to present more virulence genes than *E. faecium* [35,36,37], and our findings were consistent with previous studies. Despite the low prevalence of virulence genes in *E. faecium*, the potential for the transfer of virulence and antimicrobial-resistance genes through bacteria can be a public health problem.

## 5. Conclusions

In this study, enterococci in non-mastitic bulk tank milk from four different dairy companies showed various degrees of antimicrobial resistance and virulence-factor distribution. The enterococci demonstrated their highest rates of resistance to TET, DOX, and ERY, and the isolates were marked by the highest prevalence of resistance genes to ERY such as *erm*(B) and TET such as *tet*(L) and *tet*(M), together with transposon-gene types such as *IntTn*. Therefore, our results support the assessment that pathogens from bulk tank milk can also become a reservoir for the dissemination of antimicrobial resistance and virulence factors through cross-contamination processes.

## Figures and Tables

**Table 1:** Antimicrobial resistance of *Enterococcus faecalis* and *Enterococcus faecium* in bulk tank milk samples from four dairy companies

	No. (%) of Antimicrobial-Resistant Enterococci Isolates, by Company				
	
	A	B	C	D	Total
Antimicrobials	(n = 132) *	(n = 53)	(n = 58)	(n = 95)	(n = 338)
β-Lactams					
Penicillin	1 (0.8) _a,b_	0 (0.0) _a,b_	3 (5.2) _b_	0 (0.0) _a_	4 (1.2)
Ampicillin	2 (1.5)	1 (1.9)	0 (0.0)	0 (0.0)	3 (0.9)
Glycopeptides					
Vancomycin	0 (0.0) _a_	2 (3.8) _b_	0 (0.0) _a,b_	0 (0.0) _a,b_	2 (0.6)
Macrolides					
Erythromycin	43 (32.6) _a_	23 (43.4) _a,b_	41 (70.7) _c_	49 (51.6) _b_	156 (46.2)
Tetracyclines					
Tetracycline	96 (72.7) _a,b_	32 (60.4) _a_	51 (87.9) _c_	69 (72.6) _a,b_	248 (73.4)
Doxycycline	69 (52.3)	24 (45.3)	34 (58.6)	41 (43.2)	168 (49.7)
Fluoroquinolones					
Ciprofloxacin	3 (2.3) _a_	5 (9.4) _b_	2 (3.4) _a,b_	3 (3.2) _a,b_	13 (3.8)
Phenicols					
Chloramphenicol	16 (12.1) _a_	16 (30.2) _b_	33 (56.9) _c_	21 (22.1) _a,b_	86 (25.4)
Ansamycins					
Rifampin	29 (22.0)	12 (22.6)	10 (17.2)	20 (21.1)	71 (21.0)

* n = number of *E. faecalis* and *E. faecium* isolated from bulk tank milk, by company. Values within a column not having the same subscript letter (a, b, c) differ significantly (*p* < 0.05).

**Table 2:** Distribution of antimicrobial resistance genes and transposon genes in tetracycline- and erythromycin-resistant *Enterococcus faecalis* and *Enterococcus faecium* in bulk tank milk from four dairy companies

Genes	No. (%) of Isolates with Antimicrobial Resistance Gene(s) and Transposon Gene(s), by Company				
	A	B	C	D	Total
	(n = 40) *	(n = 18)	(n = 45)	(n = 43)	(n = 146)
*erm*(B)	2 (5.0)	0 (0)	0 (0)	0 (0)	2 (1.4)
*tdnX*	0 (0)	0 (0)	0 (0)	0 (0)	0 (0)
*erm*(B) *+ tet*(O)	0 (0)	1 (5.6)	0 (0)	0 (0)	1 (0.7)
*erm*(B) *+ tet*(L)	2 (5.0)	0 (0)	2 (4.4)	0 (0)	4 (2.7)
*erm*(B) *+ tet*(M)	3 (7.5)	0 (0)	5 (11.1)	2 (4.7)	10 (6.8)
*erm*(B) *+ IntTn*	1 (2.5)	0 (0)	0 (0)	0 (0)	1 (0.7)
*tet*(L) *+ tet*(M)	1 (2.5)	0 (0)	1 (2.2)	0 (0)	2 (1.4)
*tet*(L) *+ tet*(M) *+ IntTn*	0 (0)	1 (5.6)	1 (2.2)	0 (0)	2 (1.4)
*erm*(B) *+ tet*(O) *+ IntTn*	0 (0)	1 (5.6)	0 (0)	0 (0)	1 (0.7)
*erm*(B) *+ erm*(A) *+ tet*(L) *+ tet*(M)	0 (0)	1 (5.6)	0 (0)	0 (0)	1 (0.7)
*erm*(B) *+ tet*(L) *+ tet*(M)	6 (15.0) _a,b_	6 (33.3) _b_	6 (13.3) _a,b_	2 (4.7) _a_	20 (13.7)
*erm*(B) *+ tet*(L) *+ IntTn*	0 (0)	1 (5.6)	0 (0)	0 (0)	1 (0.7)
*erm*(B) *+ tet*(M) *+ IntTn*	20 (50.0) _a_	2 (11.1) _b_	22 (48.9) _a_	6 (14.0) _b_	50 (34.2)
*erm*(B) *+ tet*(L) *+ tet*(M) *+ IntTn*	5 (12.5) _a_	5 (27.8) _a_	8 (17.8) _a_	32 (74.4) _b_	50 (34.2)
None	0 (0)	0 (0)	0 (0)	1 (2.3)	1 (0.7)

* n = no. of tetracycline and erythromycin-resistant *E. faecalis* and *E. faecium* isolates from samples of bulk tank milk, by company. Values within a column not having the same subscript letter (a,b) differ significantly (*p* < 0.05).

**Table 3:** Distribution of virulence genes in 305 isolates of *Enterococcus faecalis* and 33 *Enterococcus faecium* in bulk tank milk from 4 dairy companies

	No. (%) of *E. faecalis* Isolates Carrying the Gene, by Company					No. (%) of *E. faecium* Isolates Carrying the Gene, by Company					*E. faecalis* vs. *E. faecium*
		
	A	B	C	D	Total	A	B	C	D	Total	
Virulence Genes	(n = 54) *	(n = 39)	(n = 88)	(n = 124)	(n = 305)	(n = 8)	(n = 14)	(n = 4)	(n = 8)	(n = 33)	*p* Value
*ace*	54 (100)	37 (94.9)	87 (98.9)	124 (100)	302 (99.0)	0 (0.0)	3 (21.4)	1 (25.0)	0 (0.0)	4 (12.1)	>0.001
*asa1*	38 (70.4) _b_	24 (61.5) _a,b_	41 (46.6)_a_	59 (47.6) _a_	162 (53.1)	0 (0.0)	2 (14.3)	1 (25.0)	0 (0.0)	3 (9.1)	>0.001
*cad1*	54 (100)	39 (100)	86 (97.7)	113 (91.1)	292 (95.7)	6 (75.0)	12 (85.7)	3 (75.0)	6 (75.0)	27 (81.8)	0.002
*cylA*	8 (14.8) _b_	4 (10.3) _b_	7 (8.0)_b_	1 (0.8) _a_	20 (6.6)	0 (0.0)	0 (0.0)	0 (0.0)	0 (0.0)	0 (0.0)	0.241
*efaA*	54 (100)	35 (89.7)	87 (98.9)	122 (98.4)	298 (97.7)	0 (0.0)	3 (21.4)	1 (25.0)	0 (0.0)	4 (12.1)	>0.001
*esp*	2 (3.7)	6 (15.4)	16 (18.2)	22 (17.7)	46 (15.1)	0 (0.0)	1 (7.1)	0 (0.0)	0 (0.0)	1 (3.0)	0.064
*gelE*	46 (85.2) _a,b_	31 (79.5) _a_	68 (77.3) _a_	117 (94.4) _b_	262 (85.9)	0 (0.0)	2 (14.3)	0 (0.0)	0 (0.0)	2 (6.1)	>0.001
*hyl*	0 (0.0)	0 (0.0)	0 (0.0)	0 (0.0)	0 (0.0)	0 (0.0)	0 (0.0)	0 (0.0)	0 (0.0)	0 (0.0)	-

* n = no. of *E. faecalis* or *E. faecium* isolates from bulk tank milk, by company. Values within a column not having the same subscript letter (a,b) differ significantly (*p* < 0.05). -; No statistics were computed because *hyl* is a constant.

## Data Availability

Not applicable.

## References

[1] Nam H.M., Lim S.K., Moon J.S., Kang H.M., Kim J.M., Jang K.C., Kim J.M., Kang M.I., Joo Y.S., Jung S.C. (2010). Antimicrobial Resistance of Enterococci Isolated from Mastitic Bovine Milk Samples in Korea. Zoonoses Public Health.

[2] Choi J.M., Woo G.J. (2015). Transfer of Tetracycline Resistance Genes with Aggregation Substance in Food-Borne Enterococcus faecalis. Curr. Microbiol..

[3] Yang F., Zhang S., Shang X., Wang X., Yan Z., Li H., Li J. (2019). Short communication: Antimicrobial resistance and virulence genes of Enterococcus faecalis isolated from subclinical bovine mastitis cases in China. J. Dairy Sci..

[4] Kim Y.B., Seo K.W., Shim J.B., Son S.H., Noh E.B., Lee Y.J. (2019). Molecular characterization of antimicrobial-resistant Enterococcus faecalis and Enterococcus faecium isolated from layer parent stock. Poult. Sci..

[5] National Institute of Food and Drug Safety Evaluation (NIFDS) (2019). National Antimicrobial Resistance Surveillance on the Domestic and Imported Meat and Fishery Products.

[6] Chung Y.S., Kwon K.H., Shin S., Kim J.H., Park Y.H., Yoon J.W. (2014). Characterization of veterinary hospital-associated isolates of Enterococcus species in Korea. J. Microbiol. Biotechnol..

[7] Kim H.J., Koo M. (2020). Occurrence, Antimicrobial Resistance and Molecular Diversity of *Enterococcus faecium* in Processed Pork Meat Products in Korea. Foods.

[8] Mannu L., Paba A., Daga E., Comunian R., Zanetti S., Duprè I., Sechi L.A. (2003). Comparison of the incidence of virulence determinants and antibiotic resistance between Enterococcus faecium strains of dairy, animal and clinical origin. Int. J. Food Microbiol..

[9] Chajęcka-Wierzchowska W., Zadernowska A., Łaniewska-Trokenheim Ł. (2017). Virulence factors of Enterococcus spp. presented in food. LWT Food Sci. Technol..

[10] Jiménez E., Ladero V., Chico I., Maldonado-Barragán A., López M., Martín V., Fernández L., Fernández M., Álvarez M.A., Torres C. (2013). Antibiotic resistance, virulence determinants and production of biogenic amines among enterococci from ovine, feline, canine, porcine and human milk. BMC Microbiol..

[11] Li X., Alvarez V., Harper W.J., Wang H.H. (2011). Persistent, toxin-antitoxin system-independent, tetracycline resistance-encoding plasmid from a dairy Enterococcus faecium Isolate. Appl. Environ. Microbiol..

[12] Ministry of Food and Drug Safety (MFDS) (2018). Processing Standards and Ingredient Specifications for Livestock Products.

[13] Dutka-Malen S., Evers S., Courvalin P. (1995). Detection of glycopeptide resistance genotypes and identification to the species level of clinically relevant enterococci by PCR. J. Clin. Microbiol..

[14] Clinical and Laboratory Standards Institute (CLSI) (2019). M100 Performance Standards for Antimicrobial Susceptibility Testing.

[15] Sweeney M.T., Lubbers B.V., Schwarz S., Watts J.L. (2018). Applying definitions for multidrug resistance, extensive drug resistance and pandrug resistance to clinically significant livestock and companion animal bacterial pathogens. J. Antimicrob. Chemother..

[16] Di Cesare A., Pasquaroli S., Vignaroli C., Paroncini P., Luna G.M., Manso E., Biavasco F. (2014). The marine environment as a reservoir of enterococci carrying resistance and virulence genes strongly associated with clinical strains. Environ. Microbiol. Rep..

[17] Agersø Y., Pedersen A.G., Aarestrup F.M. (2006). Identification of Tn5397-like and Tn916-like transposons and diversity of the tetracycline resistance gene tet(M) in enterococci from humans, pigs and poultry. J. Antimicrob. Chemother..

[18] Choi J.M., Woo G.J. (2013). Molecular characterization of high-level gentamicin-resistant Enterococcus faecalis from chicken meat in Korea. Int. J. Food Microbiol..

[19] Gao X., Fan C., Zhang Z., Li S., Xu C., Zhao Y., Han L., Zhang D., Liu M. (2019). Enterococcal isolates from bovine subclinical and clinical mastitis: Antimicrobial resistance and integron-gene cassette distribution. Microb. Pathog..

[20] Okamoto E., Miyanishi H., Nakamura A., Kobayashi T., Kobayashi N., Terawaki Y., Nagahata H. (2018). Bacteriological evaluation of composted manure solids prepared from anaerobic digested slurry for hygienic recycled bedding materials for dairy cows. Anim. Sci. J..

[21] Nam H., Lim S., Kang H., Kim J., Moon J., Jang K., Joo Y., Kang M., Jung S. (2009). Antimicrobial resistance of streptococci isolated from mastitic bovine milk samples in Korea. J. Vet. Diagn. Investig..

[22] Kim S.-J., Lee N.Y., Song J.-H., Kim S., Peck K.R., Choi M.S., Kim E.C., Lee W.G., Lee K., Pai C.H. (1998). A Study on Molecular Epidemiology of Vancomycin-Resistant Enterococci Isolated from hospitals in Korea. Korean J. Infect. Dis..

[23] Sahlström L., Rehbinder V., Albihn A., Aspan A., Bengtsson B. (2009). Vancomycin resistant enterococci (VRE) in Swedish sewage sludge. Acta Vet. Scand..

[24] Kotzamanidis C., Zdragas A., Kourelis A., Moraitou E., Papa A., Yiantzi V., Pantelidou C., Yiangou M. (2009). Characterization of vanA-type enterococcus faecium isolates from urban and hospital wastewater and pigs. J. Appl. Microbiol..

[25] Song J.Y., Hwang I.S., Eom J.S., Cheong H.J., Bae W.K., Park Y.H., Kim W.J. (2005). Prevalence and molecular epidemiology of vancomycin-resistant enterococci (VRE) strains isolated from animals and humans in Korea. Korean J. Intern. Med..

[26] Różańska H., Piłat A.L.-, Kubajka M., Weiner M. (2019). Occurrence of enterococci in mastitic cow’ s milk and their antimicrobial resistance. J. Vet. Res..

[27] Ayobami O., Willrich N., Reuss A., Eckmanns T., Markwart R.E. (2020). The ongoing challenge of vancomycin-resistant *Enterococcus faecium* and *Enterococcus faecalis* in Europe: An epidemiological analysis of bloodstream infections. Emerg. Microbes Infect..

[28] Manson J.M., Keis S., Smith J.M.B., Cook G.M. (2003). A clonal lineage of VanA-type Enterococcus faecalis predominates in vancomycin-resistant enterococci isolated in New Zealand. Antimicrob. Agents Chemother..

[29] Wang Y., Lv Y., Cai J., Schwarz S., Cui L., Hu Z., Zhang R., Li J., Zhao Q., He T. (2015). A novel gene, optrA, that confers transferable resistance to oxazolidinones and phenicols and its presence in Enterococcus faecalis and Enterococcus faecium of human and animal origin. J. Antimicrob. Chemother..

[30] Yoon S., Kim Y.B., Seo K.W., Ha J.S., Noh E.B., Lee Y.J. (2020). Characteristics of linezolid-resistant Enterococcus faecalis isolates from broiler breeder farms. Poult. Sci..

[31] Pankey G., Ashcraft D., Patel N. (2005). In vitro synergy of daptomycin plus rifampin against Enterococcus faecium resistant to both linezolid and vancomycin. Antimicrob. Agents Chemother..

[32] Huys G., D’Haene K., Collard J.M., Swings J. (2004). Prevalence and Molecular Characterization of Tetracycline Resistance in Enterococcus Isolates from Food. Appl. Environ. Microbiol..

[33] Hollenbeck B.L., Rice L.B. (2012). Intrinsic and acquired resistance mechanisms in enterococcus. Virulence.

[34] Archimbaud C., Shankar N., Forestier C., Baghdayan A., Gilmore M.S., Charbonné F., Joly B. (2002). In vitro adhesive properties and virulence factors of Enterococcus faecalis strains. Res. Microbiol..

[35] Golob M., Pate M., Kušar D., Dermota U., Avberšek J., Papić B., Zdovc I., Bondi M. (2019). Antimicrobial Resistance and Virulence Genes in Enterococcus faecium and Enterococcus faecalis from Humans and Retail Red Meat. Biomed. Res. Int..

[36] Strateva T., Atanasova D., Savov E., Petrova G., Mitov I. (2016). Incidence of virulence determinants in clinical Enterococcus faecalis and Enterococcus faecium isolates collected in Bulgaria. Braz. J. Infect. Dis..

[37] Ferguson D.M., Talavera G.N., Hernández L.A.R., Weisberg S.B., Ambrose R.F., Jay J.A. (2016). Virulence Genes among Enterococcus faecalis and Enterococcus faecium Isolated from Coastal Beaches and Human and Nonhuman Sources in Southern California and Puerto Rico. J. Pathog..

